# Untargeted metabolomics data in the By-Band-Sleeve trial and ALSPAC: integrating clinical trial and population cohort data

**DOI:** 10.12688/wellcomeopenres.24917.1

**Published:** 2025-11-10

**Authors:** Madeleine Smith, Lucy Goudswaard, David Hughes, Jane Blazeby, Chris Rogers, Graziella Mazza, Eleanor Gidman, Sophie FitzGibbon, Alix Groom, Susan Ring, Kate Northstone, Sarah Matthews, Gail White, Laurie Fabian, Nicholas Timpson, Laura Corbin

**Affiliations:** 1 Population Health Sciences, University of Bristol, Bristol, England, UK; 2 MRC Integrative Epidemiology Unit, University of Bristol, Bristol, England, UK; 3 Population and Public Health Sciences, Pennington Biomedical Research Center, Baton Rouge, Louisiana, USA; 4 Population Health Sciences and Bristol Biomedical Research Centre, University of Bristol, Bristol, UK; 5 University Hospitals Bristol and Weston NHS Foundation Trust, Bristol, England, UK; 6 Bristol Medical School, University of Bristol, Bristol, UK

**Keywords:** ALSPAC, By-Band-Sleeve, metabolomics, Metabolon, obesity, body mass index, mass-spectrometry, bariatric surgery, metabolic surgery

## Abstract

Metabolomics is the study of measured metabolites and low-molecular weight molecules in a biological specimen, collectively known as the metabolome. Measuring the metabolome in populations is useful for investigating complex, polygenic and multifactorial traits as it can give insight into cellular metabolism and its perturbations in various states of health and disease. The Avon Longitudinal Study of Parents and Children (ALSPAC) is a birth cohort that enrolled pregnant women in 1991/92 and has followed them up ever since. Samples for this work were collected from ALSPAC participants at 30 years of age. By-Band-Sleeve (BBS) is a pragmatic randomised controlled trial (RCT) which evaluated the clinical effectiveness of three different types of bariatric surgery. Two sample collection efforts were made within BBS – firstly, from the RCT comparing the effectiveness of three types of bariatric surgery: the Roux-en-Y gastric bypass (“bypass”), laparoscopic adjustable gastric band (“band”) and the sleeve gastrectomy (“sleeve”), and secondly from a non-randomised (observational) study of bariatric surgery. In both instances, samples were collected from patients before and after surgery. Samples were sent for mass-spectrometry (MS) metabolomics analysis by Metabolon (Discovery HD4 platform). Bringing together samples from these two studies in a single experiment offers a novel study design able to explore the biological implications of – in this case – weight and intentional weight loss. Data underwent quality control (QC) via a standard pipeline using the R package *metaboprep*. Post-filtering, the dataset consists of semi-quantitative data for 1253 features in 517 ALSPAC participants and 1062 BBS participants (1018 from the RCT and 44 from the non-randomised study) (1013 pre-surgery samples and 421 post-surgery samples). Overall, we have provided a summary of MS data produced across two different study populations, described the QC procedures undertaken and provided some data validation analyses.

## Introduction

Metabolomics is the study of measured metabolites and low-molecular weight molecules in a biological specimen, collectively known as the metabolome. Metabolomics enables detailed insight into cellular metabolism and its perturbations in various states of health and disease ^1, 2^. Variation in the metabolome has genetic, lifestyle, environmental, microbial and pharmacological underpinnings and therefore metabolomics within large-scale studies is useful for investigating complex, polygenic and multifactorial traits ^3^. Here we present a description of metabolomics data generated using an untargeted mass-spectrometry approach in two studies: (1) the Avon Longitudinal Study of Parents and Children (ALSPAC) - a healthy, general population; and (2) the By-Band-Sleeve trial (BBS) - a randomised controlled trial (RCT) of bariatric surgery plus a non-randomised observational sub-study.

ALSPAC is a birth cohort of children born within the Bristol (UK) area in 1991–1992 with detailed phenotypic measurements from early life to the most recent data collection conducted when the participants were around 30 years of age ( www.bristol.ac.uk/alspac) ^4– 6^. The study has also collected detailed data from the mothers and their partners throughout the same period, providing the opportunity to study parental influences on offspring phenotypes. ALSPAC is a valuable resource for health research due to the breadth of variables measured and bioresources available. This addition of metabolite data will enrich the existing ALSPAC data and enable further research into the role of metabolites in health and disease.

BBS is a trial which compared the clinical and cost-effectiveness of three types of bariatric surgery common in the UK ^7– 9^. Clinical trials of weight loss interventions such as surgery provide an opportunity to generate data able to describe the biological response to variation in weight, which is known to have a broad effect on cellular metabolism ^10^. Metabolomic data in BBS will allow researchers to assess the effect of bariatric surgery and weight loss on circulating metabolites. Given the randomised design of BBS, there is also the opportunity to explore whether specific types of surgery have differential effects on subsequent metabolic health.

This data note describes the collection and processing of MS metabolomic data in 520 samples from ALSPAC and 1608 samples from BBS. It describes the source studies, sample collection, metabolite measurement and pre-analytical data processing and quality control, before discussing the potential uses of the dataset, and its strengths and limitations.

## Materials and methods

### Overview of data sources

***Avon Longitudinal Study of Parents and Children (ALSPAC)***

ALSPAC enrolled pregnant women in the Avon region surrounding Bristol (UK) between 1 ^st^ April 1991 and 31 ^st^ December 1992 ^4– 6^. A total of 14,541 pregnancies were enrolled, resulting in 14,676 foetuses, 14,062 live births and 13,988 children alive at the age of 1. When the oldest children were approximately seven years of age, an attempt was made to bolster the initial sample with eligible cases who had failed to join the study originally. As a result, when considering variables collected from the age of seven onwards (and potentially abstracted from obstetric notes) there are data available for more than the 14,541 pregnancies mentioned above. The number of new pregnancies not in the initial sample (known as Phase I enrolment) that are currently represented in the released data and reflecting enrolment status at the age of 24 is 906, resulting in an additional 913 children being enrolled (456, 262 and 195 recruited during Phases II, III and IV respectively). The phases of enrolment are described in more detail in the cohort profile paper and its update ^6^. The total sample size for analyses using any data collected after the age of seven is therefore 15,447 pregnancies, resulting in 15,658 foetuses. Of these 14,901 children were alive at 1 year of age. The study website contains details of all the data that is available through a fully searchable data dictionary and variable search tool ( http://www.bristol.ac.uk/alspac/researchers/our-data/). Study data were collected and managed using REDCap (Research Electronic Data Capture) electronic data capture tools hosted at the University of Bristol ^11^. REDCap is a secure, web-based software platform designed to support data capture for research studies.

***By-Band-Sleeve***

BBS is an open parallel-group multi-site randomised controlled trial which took place in the UK (registration number: ISRCTN00786323) ^7^. The trial investigated the clinical and cost-effectiveness of three types of bariatric surgery: the Roux-en-Y gastric bypass (“Bypass”), laparoscopic adjustable gastric band (“Band”) and the sleeve gastrectomy (“Sleeve”). This study began in December 2012 as “By-Band” comparing Band and Bypass, the two most common bariatric surgery methods in the UK at that time. However, due to the increasing number of sleeve gastrectomy procedures taking place in the UK during the first few years of the study, Sleeve was added to the study which became “By-Band-Sleeve” in August 2015 ^8^. The pilot phase of the study occurred in two study sites (designated below as A and B), expanding into ten more sites (C-L) after the adaptation to include sleeve. Ten of the twelve sites agreed to collect and store biological samples for future research. At completion of recruitment in September 2019, 1351 patients had been randomised as part of the trial ^9^. The primary endpoint for the trial was 36-months post-randomization and follow-up to this timepoint was completed by the end of March 2023 with the main trial outcome reported in May 2025 ^12^.

*By-Band-Sleeve non-randomised metabolomics sub-study*

When recruitment to the main BBS trial concluded (September 2019), recruitment to a non-randomised metabolomics sub-study began and ran until June 2022, at which point 214 patients had been consented. Eligibility criteria remained the same as in the main trial, but participants of the sub-study were not randomised to a surgery type and instead underwent the surgical procedure as determined by the usual process at their hospital with follow-up care as standard. Participants were followed up for 12 months after their operation. Seven of the original 12 study sites opted to recruit patients for the sub-study, including site L which had not collected samples for the main trial. A reduced set of clinical data were collected for the sub-study participants, using a different data collection form to the main study that focused on weight and diabetes outcomes pre-surgery and at follow-up. Herein, the two parts of BBS described will be referred to as the main trial and the sub-study.

*By-Band-Sleeve – derived variables*

Variables were derived to capture the differences in timing of surgery and sample collection across participants. Time between baseline sample collection and surgery date, time between surgery date and follow-up sample collection, and time between baseline and follow-up sample collection were calculated in days and presented to the nearest month. Storage time was calculated as the difference between the date the sample was collected and the date it was thawed for aliquoting prior to metabolomics analysis, in days to the nearest month. Disruption by the COVID-19 pandemic meant that adherence to the BBS trial protocol (especially with respect to the format and timing of the follow-up appointments) was interrupted in some cases. For example, some of the 36-month appointments were conducted over the phone, with bloods either not collected or collected at a later date when COVID-19 restrictions would allow.

### Ethical approval

This study was conducted in accordance with the principles of the Declaration of Helsinki.

Ethical approval for ALSPAC was obtained from the ALSPAC Ethics and Law Committee and the Local Research Ethics Committees. Consent for biological samples has been collected in accordance with the Human Tissue Act (2004). Informed consent for the use of data collected via questionnaires and clinics was obtained from participants following the recommendations of the ALSPAC Ethics and Law Committee at the time. For the clinic at age 30, ethics approval was obtained from London Queen's Square research Ethics committee (reference 21/LO/0588). The use of the samples for this project (B4132) was approved by the ALSPAC Executive and Independent Scientific Advisory Board.

Ethical approval for BBS was granted by the Southwest Frenchay Research Ethics Committee (reference 11/SW/0248) and written informed consent was obtained from all participants. All samples were used, stored and disposed of in accordance with the Human Tissue Act 2004.

### Sample collection

***ALSPAC***

ALSPAC participants have been invited to attend various in-person clinics since childhood, with the most recent clinic running from October 2021 to August 2024 when participants were around 30 years of age. A small number of participants were newly recruited to the study at this time. Fasting blood samples were collected by venepuncture into 5mL serum tubes and left to clot at room temperature for 30 minutes before centrifugation at 1300g at room temperature for 10 minutes to produce serum. Participants with diabetes and on insulin or who were pregnant were instructed not to fast. The samples were stored at -80°C within 120 minutes of blood taking. Samples from 520 participants who had attended the clinic by 24 ^th^ October 2022 and had at least three aliquots of serum stored were selected for metabolomic analysis.

***By-Band-Sleeve***

During the BBS main trial, data collection points were scheduled at baseline (pre-randomisation), the day of surgery, four weeks post-surgery, and 6-, 12-, 24- and 36-months post-randomisation. Separately to the work presented here, blood samples were taken at each timepoint (excluding the day of surgery) for hospital laboratory analyses including, for example, full blood count, liver function tests and HbA1c (haemoglobin A1c, measure of glycated haemoglobin) measurement. Patients were asked to fast for a minimum of four hours prior to blood collection. In addition, blood samples were collected at baseline and 36-months post-randomisation and used to derive whole blood, plasma and serum samples for future research. From this collection, serum samples were used to generate metabolomics data. Blood was collected into 4ml vacutainers containing clot activator gel (Serum Separation Tubes (SST)) and processed within one hour of collection when possible. The blood was allowed to clot for 30 minutes before centrifugation at 1500g for 10 minutes at room temperature to separate the serum. The serum was transferred into sterile polypropylene tubes with screw caps using a Pasteur pipette or similar and stored at -80°C before being shipped to the coordinating centre (University of Bristol) on dry ice.

In the BBS non-randomised sub-study, fasting blood samples were collected pre-surgery and 12 months post-surgery and used to derive serum, following the same protocol as the main trial. The serum was transferred into up to four sterile polypropylene tubes with screw caps with a target sample volume of 0.5ml per tube. These blood samples were stored at -80°C before being transferred from each recruiting site to the University of Bristol for storage.

From the main trial, 1518 samples from 1149 participants collected between January 2013 and June 2022 were sent for metabolomic analysis. These were all the samples available in Bristol at the point of shipping; not all participants had both a baseline and a 36-month sample. From the sub-study, 45 pairs of samples (90 samples) collected between October 2019 and May 2022 were sent for metabolomics analysis; these were all the sample pairs available in Bristol at the point of shipping. The total number of BBS samples (main trial and sub-study) analysed was 1608. The number of baseline, endpoint and paired samples differed across the sites ( Table 1). All ALSPAC and BBS samples were thawed for the first time since collection upon aliquoting samples for shipment.

**Table 1.** The distribution of BBS samples across the 12 study sites (main trial and non-randomised sub-study).

Site	Samples	Baseline samples	Endpoint samples	Number of pairs
**A**	182	144	38	34
**B**	447	258	189	184
**C**	65	49	16	14
**D**	82	66	16	15
**E**	0	0	0	0
**F**	159	93	66	64
**G**	196	163	33	31
**H**	135	113	22	22
**I**	84	82	2	2
**J**	71	55	16	16
**K**	163	140	23	21
**L**	22	11	11	11
**TOTAL**	1606	1174	432	414

### Metabolite data acquisition

A total of 2128 serum samples from ALSPAC and BBS were sent to Metabolon to be analysed as a single experiment. The mass spectrometry platform used by Metabolon to measure metabolites provides semi-quantitative abundance data rather than absolute quantifications; therefore, the data are relative and cannot be combined across studies at the individual level after metabolomics analysis. Having ALSPAC and BBS samples analysed within the same experiment allows the data to be analysed together.

Untargeted metabolomic measurements were performed using the Discovery HD4 platform. Here, ultra-high-performance liquid chromatography/tandem accurate mass spectrometry was used, with references to libraries of biochemicals based on standards with mass-to-charge ration ( *m/z*), retention time/index and chromatographic data. The laboratory process requires samples to be organised into sets of 36 samples, each of which represent an analytical batch. To avoid confounding of analytical batch with source study or timepoint, samples were randomized into 60 sets of 36 whilst accounting for source study and timepoint. Specifically, samples from each of the three studies (ALSPAC, BBS main trial and BBS sub-study) were balanced across the sets of 36 and sample pairs from the same participants (baseline and endpoint) were kept together in the same set. The 32 empty slots were equally distributed across sets. Batch information is available and can be provided alongside the metabolite data.

Methodological details provided by Metabolon can be found in Supplementary File 1 – Extended methods in Extended Data and refer to work undertaken by them. The dataset was downloaded from the Metabolon server on 28 ^th^ February 2023. A total of 1331 biochemicals were detected using this platform, of which 1076 had known identity and 255 were compounds whose structural identity were unknown. Identified metabolites were assigned to super pathways by Metabolon, namely, amino acids, carbohydrates, cofactors and vitamins, energy, lipids, nucleotides, partially characterised molecules, peptides and xenobiotics.

### Pre-analytical processing

Five versions of the dataset were provided: peak area data (raw data), batch-normalised data, batch-normalised and imputed (with minimum) data, log transformed data and volume-extracted normalised data. A description of the batch-normalization and imputation methods applied to the data is included in Supplementary File 1 – Extended methods in Extended Data. Here, the volume-extracted normalised version of the metabolite data was carried forward to pre-analytical processing. This was due to one of the ALSPAC samples only having an extracted volume of 90uL rather than the target volume of 100uL. The volume-extracted normalised data accounts for this difference in extracted sample volume. For each sample, the batch-normalized data are divided by the value of the normalizer. Then each metabolite is re-scaled to have median = 1 (by dividing the new values by the overall median for each metabolite). The volume-extracted normalized data provided was imputed such that missing values were replaced with the minimum observed value. Here, imputed values were removed to revert the data to their unimputed format.

Volume-normalised data underwent pre-processing and QC using the R package *metaboprep* ^13^. *Metaboprep* is an R package that provides a standard data processing workflow for metabolite data returned from specific commercial platforms. The package produces summaries of datasets and performs sample and feature (metabolite) filtering based on user-defined thresholds.

We conducted multiple implementations of the *metaboprep* pipeline to account for the structured nature of the dataset. Firstly, the entire dataset (all samples from ALSPAC, BBS main trial and BBS sub-study) was run through the *metaboprep* pipeline with default settings and results from the principal component analysis (PCA) feature used to check for any unexpected heterogeneity (substructure) in the data. Within *metaboprep*, PCA was performed using a subset of independent features. Independent features were identified by constructing a dendrogram based on a Spearman’s rho distance matrix, where distance is calculated using the absolute value of 1-Spearman’s rho. A set of k-clusters were identified using tree cut height of 0.5. Within each k-cluster, one representative feature was chosen to represent the cluster. It was identified as the feature with least missingness or chosen at random in the case of missingness ties. Only for the purpose of performing the PCA, missing values in this set of independent features were imputed to the median before the data were standardised (z-transformed) such that the mean was equal to zero and the standard deviation was equal to one for each feature. An outlying cluster of samples identified by the PCA in this run were then excluded from the dataset, before performing a second run of the pipeline, to further filter the data based on missingness, total sum abundance (TSA, the sum of abundance values across all features for a single sample) and principal components outliers.

In the second *metaboprep* run, both sample missingness and feature missingness were considered when filtering the data. Metabolites of the xenobiotic class were not included in calculations of sample missingness, because these are exogenous compounds (e.g., drugs) where missing data are more likely to represent absence of the metabolite. Xenobiotics can have high rates of missingness but still be critically informative to a study. Any samples and features with extreme missingness (>80%) were excluded before recalculating sample and feature missingness. Then samples with more than 20% missingness and features with more than 50% missingness were removed from the dataset. TSA was calculated to further assess the sample quality. Samples with a TSA five standard deviations (SDs) from the mean TSA were excluded. The PCA analysis (as described above) was repeated and any samples that were more than five SDs from the mean of the first and/or second principal components were filtered from the data set.

Although the dataset was created with joint analysis in mind, it is also possible to analyse the studies separately and therefore we provide study-specific summary statistics to go with the data. To this end, after the second *metaboprep* run, the filtered data were divided based on their source studies into two separate datasets: ALSPAC and BBS. Each dataset was then run through *metaboprep* a final time, without any exclusion parameters, and solely for the purpose of obtaining study-specific summary statistics.

### Dataset validation

Using the post-QC datasets, we performed a series of analyses designed to validate the data.

***Comparison with clinical chemistry***

We generated Bland-Altman plots (on z-scored data) and explored Pearson’s correlations to compare (batch normalised) MS data and clinical chemistry data for the metabolites that were measured by both methods. The clinical chemistry assays were performed on samples collected at the same time as the samples sent for MS analysis (i.e., at the 36-month post-randomization timepoint) and using standard procedures. The metabolites that were compared are: glucose, urea, creatinine, bilirubin and cholesterol. For glucose and cholesterol, we used clinical data from BBS main trial participants and ALSPAC, and for the other metabolites (urea, creatinine and bilirubin) we only had clinical data from BBS main trial participants (they were not measured in ALSPAC at the time of writing and BBS sub-study samples did not undergo clinical chemistry assays). In ALSPAC, glucose and cholesterol were measured using a CardioChek Professional Analyser with CHOL+GLU test strips (BIOP101 and BIOS002D; BHR Pharmaceuticals Ltd, UK). The CardioChek analyser was calibrated weekly using control solution and checked daily using test strips. In BBS, the clinical chemistry metabolites were measured at the hospital where the participant was recruited via standard NHS procedures.

***Comparison to previous BBS MS data***

250 samples from 125 site B participants in the BBS main trial were first analysed by Metabolon in June 2021, as a pilot experiment. These 250 samples were then sent for analysis again within the current BBS/ALSPAC dataset and therefore these samples have been analysed twice. When sending the samples for the second time, different aliquots from the same sampling occasion were used, to ensure that they had undergone the same number of freeze/thaw cycles. The batch-normalised data from each run (2021 vs. 2023) was compared by generating Bland-Altman plots (on z-scored data) and by calculating Spearman’s rank correlation coefficients for each metabolite.

***Batch normalization***

One nuance of this dataset is the integration of two studies that may be analysed separately or collectively, providing future users with three possible datasets (1. BBS, 2. ALSPAC, 3. BBS + ALSPAC). A complication that arises from this is choosing when to perform batch normalization. Here we have chosen to perform the normalization once using all the data as provided by Metabolon in the volume-extracted normalized data and described above. An alternative approach would be to conduct the batch normalization within the ALSPAC and BBS data sets, individually. A series of simulations were conducted to evaluate whether a within-study batch normalization approach is necessary prior to the analyses of data from a single study. Simulations assumed data are derived from a random normal distribution and that samples from ALSPAC and BBS are distributed at random within those distributions. The code for the simulations tested can be found in the study’s GitHub repository (see below).

## Results

### Pre-analytical processing by metaboprep

**Figure 1.**
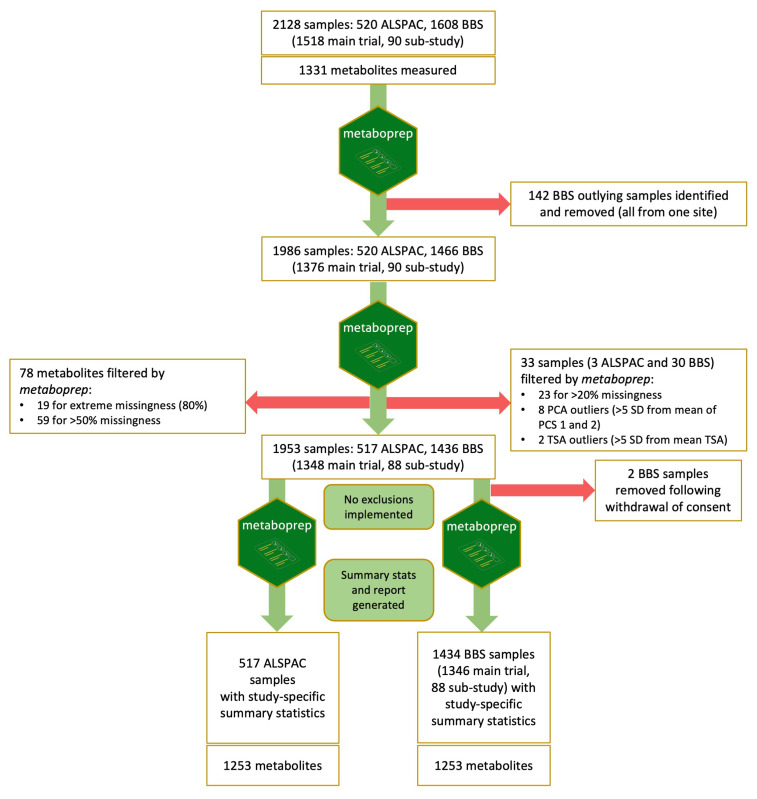
Overview of pre-analytical processing and data filtering. The R package *metaboprep* pipeline was implemented firstly to identify any unexpected sample heterogeneity, then a second time to filter the samples and metabolites based on missingness, PCs and TSA. Finally, the pipeline was run again on the full dataset and on ALSPAC and BBS datasets separately to generate study-specific summary statistics and reports. ALSPAC, Avon Longitudinal Study of Parents and Children; BBS, By-Band-Sleeve; PCA, principal component analysis; TSA, total sum abundance.

**Figure 2.**
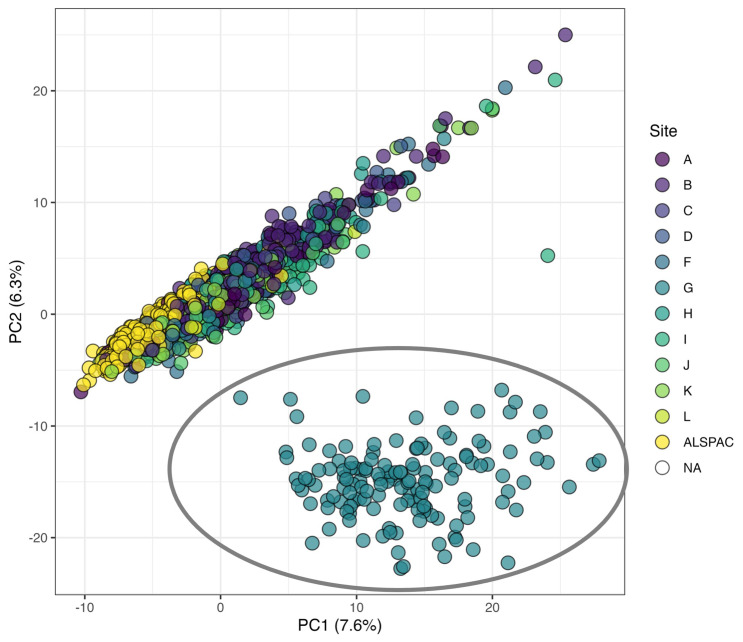
Principal component (PC) plot from the first *metaboprep* run where an outlying cluster of samples from site G were identified based on PCs 1 and 2, coloured by study site. The 142 samples within the grey oval were excluded from subsequent QC steps and statistical analyses. The axis labels indicate the variance explained (%) by each PC.

An overview of pre-analytical data processing and filtering is provided in Figure 1. During the first *metaboprep* run on the full dataset (2128 samples), visual inspection of the PCA plot of the unfiltered data identified a group of 142 outlying samples (Supplementary File 2). On further investigation, it was determined that these samples were all collected from BBS study site G, though not all the samples from site G were outliers ( Figure 2). The outlying samples tended to have been collected earlier in the study (2015–2019) than the rest of the site G samples (2019–2020) and were mostly baseline samples (137 baseline). The precise cause of this sample structure could not be determined but was deemed to be most likely a technical artefact, possibly the result of differences in sample processing, and therefore the 142 outliers were removed from the dataset prior to any further QC and filtering.

During the second *metaboprep* run on the 1986 samples remaining in the full dataset, 78 features were removed based on feature missingness (>50% missing). A total of 33 samples (3 ALSPAC and 30 BBS) were excluded: 23 based on user-defined sample missingness (>20% missing), two based on TSA, and eight excluded as PCA outliers (Supplementary File 3).

The resulting filtered dataset therefore had 1953 samples and 1253 features. Following the removal of data from two BBS participants who had subsequently withdrawn consent for their data to be used, 1951 samples remained comprising 517 ALSPAC samples from the same number of individuals and 1434 BBS samples from 1062 individuals (1018 from the main trial and 44 from the non-randomised sub-study; 1013 pre-surgery samples and 421 post-surgery samples). A metaboprep summary report provides summary statistics for the combined dataset (Supplementary File 4) and for each filtered data set (BBS and ALSPAC) separately (Supplementary Files 5 & 6).

### Participant characteristics

***ALSPAC***

The characteristics of the ALSPAC participants that had samples sent to for analysis are summarised in Table 2. Diabetes status was not available for these participants at the time of analysis, but they had been asked about insulin treatment during a data collection at age 24 years. One of the 520 participants was newly recruited during the Focus at 30 clinic.

**Table 2.** Participants characteristics for ALSPAC samples (N=520).

Characteristic	N ^1^	n (%) / mean (SD) ^2^
Sex	520	
Male		202 (39%)
Female		318 (61%)
Ethnicity	443	
British		416 (93.9%)
Other		27 (6.1%)
Insulin user (at 30y)	520	<5
Age (years)	520	29.8 (0.6)
Weight (kg)	516	76.9 (18.1)
BMI (kg/m^2)	516	25.9 (5.6)

^1^N with data. ^2^For continuous variables values are mean (SD), for categorical variables values are n (%).

***By-Band-Sleeve***

The baseline characteristics of all BBS main trial participants have been published ^9^. The characteristics of the subsample of 1147 main trial participants and the 45 sub-study participants that have MS data are described in Table 3.

**Table 3.** Participant characteristics for the BBS main trial and sub-study.

Characteristic	N ^1^	Overall, N = 1,192 ^2^	Main, N = 1,147 ^2^	Sub-study, N = 45 ^2^
Sex	1,167			
Male		285 (24%)	279 (25%)	6 (13%)
Female		882 (76%)	843 (75%)	39 (87%)
Age (years)	1,166	47 (11)	47 (11)	45 (10)
Ethnicity	1,167			
White		1,008 (86%)	963 (86%)	45 (100%)
Mixed/multiple ethnic groups		25 (2.1%)	25 (2.2%)	<5
Asian/Asian British		58 (5.0%)	58 (5.2%)	<5
Black/African/Caribbean/Black British		65 (5.6%)	65 (5.8%)	<5
Other		11 (0.9%)	11 (1.0%)	<5
Diabetes status	1,167			
No indication		811 (69%)	773 (69%)	38 (84%)
Glucose impairment		13 (1.1%)	13 (1.2%)	<5
Oral hypoglycaemics		227 (19%)	223 (20%)	<5
Insulin		56 (4.8%)	56 (5.0%)	<5
GLP-1 agonist		25 (2.1%)	23 (2.0%)	<5
Diet controlled		35 (3.0%)	34 (3.0%)	<5
Baseline ^3^ weight (kg)	1,167	130 (23)	130 (24)	126 (21)
End ^4^ weight (kg)	1,011	103 (26)	103 (26)	193 (22)
Baseline ^3^ BMI (kg/m ^2^)	1,167	47 (7)	47 (7)	46 (6)
End ^4^ BMI (kg/m ^2^)	1,011	37 (8)	37 (8)	34 (6)
Diabetes duration (months) ^5^	313	74 (70)	74 (71)	43 (34)

^1^N with data. ^2^For continuous variables values are mean (SD), for categorical variables values are n (%). ^3^’Baseline’ refers to the first pre-surgery measures; these were taken at randomization in the main trial and at the first visit in the non-randomised sub-study. ^4^’End’ refers to the measures taken at 36-months post-randomization in the main trial and at 12-months post-surgery in the non-randomised sub-study. ^5^Diabetes duration at the time of recruitment. <5 may include zero.

### Dataset validation

Using the post-QC datasets, we performed a series of analyses designed to validate the data.

***Comparison with clinical chemistry***

**Figure 3.**
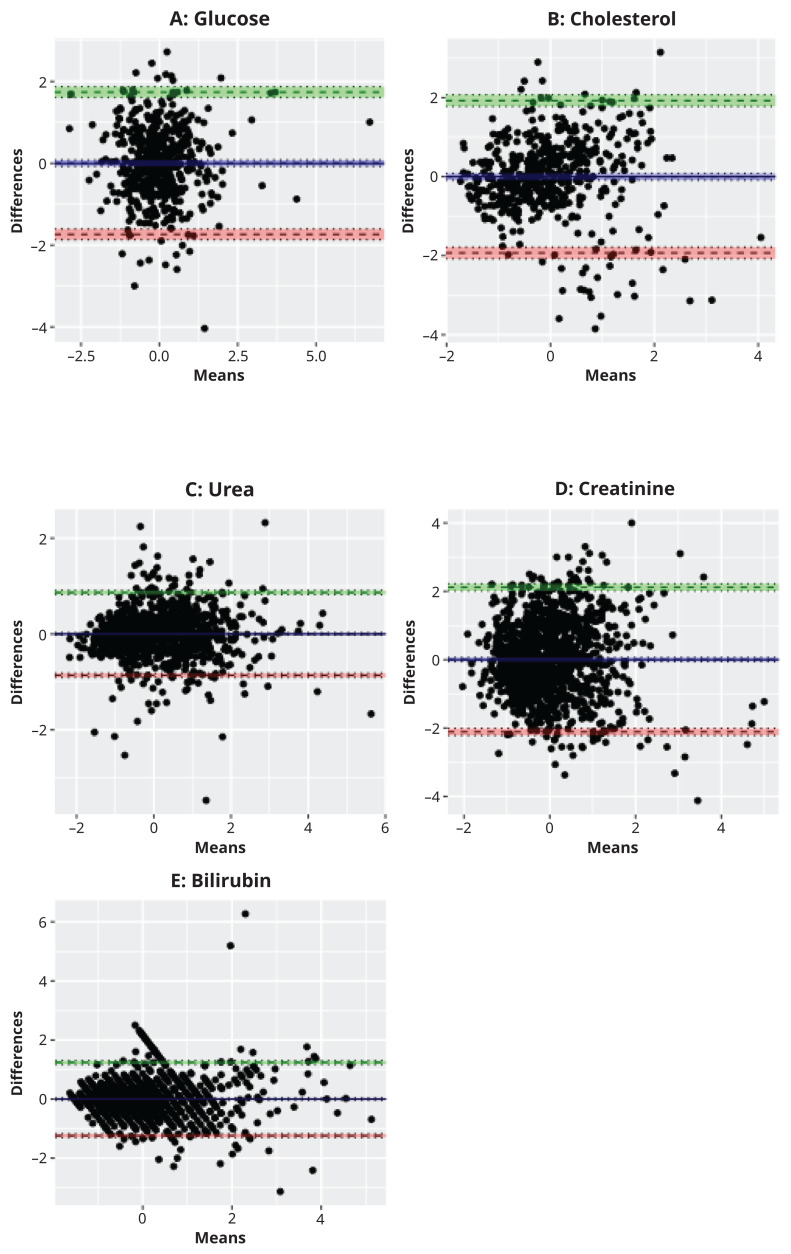
Bland-Altman plot comparison of ( **A**) glucose (n=1784), ( **B**) cholesterol (n=1837), ( **C**) urea (n=1316), ( **D**) creatinine (n=1314) and ( **E**) bilirubin (n=1310) concentrations derived from MS and clinical chemistry; ALSPAC, Avon Longitudinal Study of Parents and Children; BBS, By-Band-Sleeve.

We explored correspondence between MS-derived and clinical chemistry-derived measures for the metabolites that were measured by both methods ( Figure 3 and Supplementary Figure 1). One outlier was removed from the clinical fasting glucose variable in BBS before plotting and calculating correlation coefficients as it was assumed to be a reporting error (76 mmol/L). The MS data mostly correlated with the clinical chemistry assays well (range *r*=0.61 – 0.90) (Supplementary Figure 1), with the exception of cholesterol which had a Pearson’s correlation coefficient of 0.51 (95% confidence interval: 0.44, 0.57) and 0.56 (95% CI: 0.52, 0.59) in ALSPAC and BBS, respectively. Bias was difficult to assess due to the semi-quantitative nature of the MS data (and the need to scale prior to Bland-Altman analysis) but there was little evidence for heteroscedasticity ( Figure 3).

***Comparison to previous BBS MS data***

**Figure 4.**
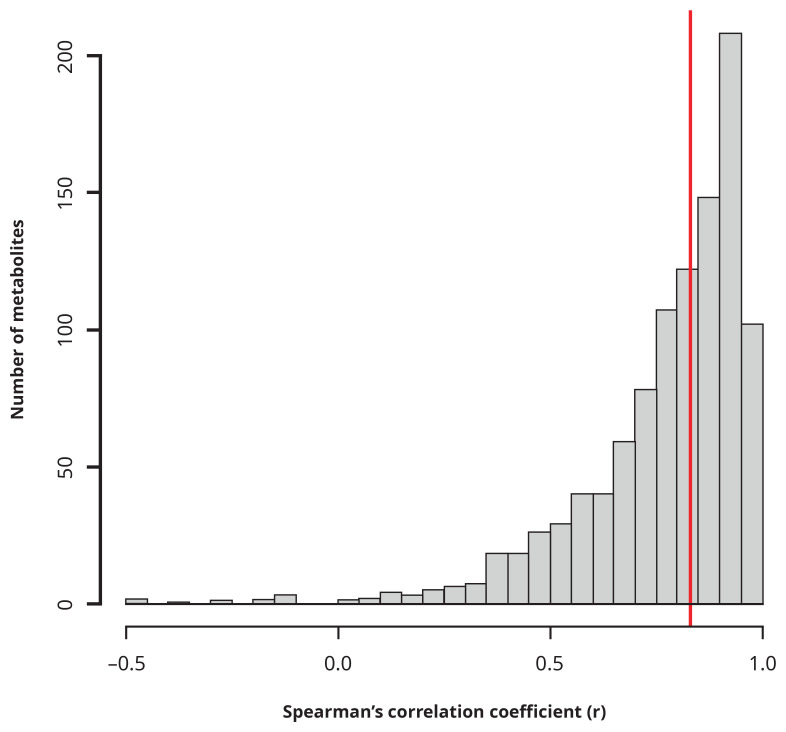
Histogram of Spearman's correlation coefficients per metabolite for data derived in June 2021 versus that data derived in February 2023, for a subset of samples from BBS site B. Red line is the median.

The batch-normalised data from each run (2021 vs. 2023) was compared using Bland-Altman plots (Supplementary Figure 2) and by calculating the Spearman’s rank correlation coefficients for each metabolite ( Figure 4). Overall, we observed a median *r* of 0.83 and an interquartile range of 0.22. However, some metabolites did not correlate well across the two runs (94/1029 metabolites had an *r* < 0.5, six had negative *r* estimates) and should therefore be treated with caution in future analyses. Of these, 27 are in the lipid class, 14 are in the xenobiotic class and 18 are in the amino acid class. High missingness appears to account for some of the low correlations, with four of the six metabolites with negative *r* estimates having >90% missingness; in addition, there was evidence (from Bland-Altman) for heteroscedasticity affecting the remaining two metabolites (with negative correlations). We provide a table of the correlation coefficients and p-values for each metabolite in Table S1.

***Batch normalization***

Based on simulations, we find that extracting ALSPAC and BBS sample populations (i.e., sub-sampling from median normalized batches) creates little to no additional noise to the sub-sampled data. In contrast, if we sub-sampled (i.e., extracted study-specific datasets) first and then median normalized we found that as batch sample sizes decrease, the noise introduced by batch normalization only increases. This observation is true when assuming there was no batch effect or when simulating batch effects. In short, noise is decreased by keeping batches as large as possible. This suggests that by balancing samples from the different studies across batches and proceeding with the provider’s standard normalisation procedure we have produced two datasets that can be analysed both independently and together. However, it is worth noting that metabolite data here are not normally distributed, BBS and ALSPAC samples are not expected to be distributed at random within a metabolite distribution, and there is the added complication of missing data. As a result, if a researcher wishes to perform an analysis within a study (ALSPAC or BBS) that compares abundance levels across metabolites a centring of the data by feature (metabolite) would be advisable. In addition, given the observed kurtosis and skew in the distribution of metabolites an inverse rank normal transformation would be advisable for all parametric analyses.

## Discussion

In this data note we have given an overview of MS metabolite data for 520 samples collected at the ALSPAC Focus at 30 clinic and 1608 samples from BBS participants. We have provided details of the pre-analytical data processing, QC, participant characteristics and data validation of these data. When comparing the metabolites in common across MS and clinical chemistry assays, we found good overall concordance although there were some outlying samples. We recommend researchers using the data consider these potential outliers before commencing analyses, and deal with them in a way that is appropriate to their research question. The lowest cross platform correlation was for total cholesterol; this may be because the two technologies are measuring slightly different lipid entities and with differential sensitivity and specificity. Comparing data from a subset of samples that we had previously generated MS data for highlighted that some metabolites were not consistently measured across all concentrations. Users may want to check that any metabolites of interest in their analyses have good consistency according to these results.

The availability of the MS data at age 30 years will be a valuable resource to add to the ALSPAC collection. The combination of these metabolite data with other questionnaire and clinic data will enable a host of research questions to be explored. MS metabolomics data are already available for a subset of ALSPAC participants who were retrospectively included in a recall-by-genotype study investigating the effect of BMI on metabolites, though this subset of participants were selected based on polygenic risk for high/low BMI so are not representative of the whole cohort ^14^. The ALSPAC participants in the present dataset were not selected based on any specific characteristics and therefore should be broadly generalisable to the whole cohort.

The metabolomic data in BBS presents a wealth of opportunity to explore the biological mechanisms that underpin how bariatric surgery influences weight loss and health. Further, BBS can be compared to populations undergoing different weight loss interventions such as caloric restriction, allowing metabolomic perturbations resulting from weight-loss to be differentiated from intervention-specific effects.

### Strengths and limitations

A key strength of the dataset is that both source studies are well-characterised, which will enable investigation of the metabolomic effects of a range of phenotypes. However, ALSPAC and BBS are very different studies comprising different populations and delivered according to different protocols, therefore the combined data should be used with caution. Further, the pre-processing and QC procedures were conducted on the combined dataset and further QC on the separate datasets may be required for those using either the ALSPAC or the BBS data in isolation. Transformation of the data may also be required before analysis to meet various statistical assumptions. The participants of both ALSPAC and BBS are predominantly White and of European ancestry and therefore the data are limited in their generalisability to other populations beyond the UK. Furthermore, the ALSPAC participants in this dataset are those that attended the age 30 years clinic first and as early attenders these people may have specific characteristics. Many of the BBS baseline (pre-surgery) samples do not have a post-surgery sample from the same participant, which reduces the power of the data to assess metabolomic change associated with bariatric surgery. However, the unpaired baseline samples can still contribute to statistical models.

## Conclusion

Overall, we have provided a summary of MS data produced across two different study populations, described the QC procedures undertaken and provided some data validation analyses. Future users of the data may wish to apply additional QC procedures and validation analyses. The filtered dataset consists of 1951 samples with untargeted mass-spectrometry metabolomics data alongside comprehensive clinical characteristics. The dataset provides a useful tool for exploring the metabolomic footprint of bariatric surgery (BBS), with the opportunity for comparison to metabolomic profiles in a healthy population (ALSPAC). Furthermore, the ALSPAC data can be used as a separate metabolomic dataset alongside the cohort’s detailed phenotypic data.

## Data Availability

Three datasets have been generated as part of this work: A joint ALSPAC and BBS dataset consisting of 1951 samples. A BBS-only dataset consisting of 1434 samples (1346 main trial and 88 non-randomised sub-study). An ALSPAC-only dataset consisting 517 samples. ALSPAC data access is through a system of managed open access. The steps below highlight how to apply for access to the data included in this data note and all other ALSPAC data. The datasets presented in this article are linked to ALSPAC project number B4132, please quote this project number during your application. 1. Please read the ALSPAC access policy ( www.bristol.ac.uk/media-library/sites/alspac/documents/researchers/data-access/ALSPAC_Access_Policy.pdf) which describes the process of accessing the data and samples in detail, and outlines the costs associated with doing so. 2. You may also find it useful to browse our fully searchable research proposals database ( https://proposals.epi.bristol.ac.uk/?q=proposalSummaries), which lists all research projects that have been approved since April 2011. 3. Please submit your research proposal ( https://www.bristol.ac.uk/alspac/researchers/access/) for consideration by the ALSPAC Executive Committee. You will receive a response within 10 working days to advise you whether your proposal has been approved. If you have any questions about accessing data, please email alspac-data@bristol.ac.uk. Anonymised individual patient data from the By-Band-Sleeve trial will be made available upon request to the chief investigator for secondary research, conditional on assurance from the secondary researcher that the proposed use of the data is compliant with the Medical Research Council Policy on Data Sharing regarding scientific quality, ethical requirements, and value for money, and is compliant with the National Institute for Health and Care Research policy on data sharing. A minimum requirement with respect to scientific quality will be a publicly available prespecified protocol describing the purpose, methods, and analysis of the secondary research (e.g., a protocol for a Cochrane systematic review), approved by a UK Research Ethics Committee or other similar, approved ethics review body. Participant identifiers will not be passed on to any third party. Open Science Framework: Supplementary information supporting this submission can be found on the Open Science Framework “Metabolomics data in the By-Band-Sleeve trial and ALSPAC: integrating clinical trial and population cohort data” project page, https://osf.io/7ptf8/ (DOI: 10.17605/OSF.IO/7PTF8). This project contains the following extended data: ‘SF1-Extended_methods.docx’ contains text provided by Metabolon to describe metabolite data acquisition. ‘SF2-metaboprep1_summary_report.html’ is the first *metaboprep* summary report. ‘SF3-metaboprep2_summary_report.html’ is the second *metaboprep* summary report, after outlying samples were removed. ‘SF4-metaboprep3_all_summary_report.html’ is the *metaboprep* summary report summarising the complete filtered dataset. ‘SF5-metaboprep3_bbs_summary_report.html’ is the *metaboprep* summary report summarising the BBS samples in the filtered dataset. ‘SF6-metaboprep3_alspac_summary_report.html is the *metaboprep* summary report summarising the ALSPAC samples in the filtered dataset. ‘SF7-figureS1.pdf – contains scatter plots of clinical versus MS measures for the five metabolites in both datasets. ‘SF8-figureS2.pdf’ - contains Bland-Altman plots for each metabolite comparing the 2022 pilot analysis to the 2023 analysis, for a subset of 250 samples collected at site B. ‘SF9-tableS1.xlsx’ – Table S1 that contains results of the correlation analysis comparing data derived in 2021 and 2023 for the same samples. Data are available under the terms of the Creative Commons Attribution 4.0 International license (CC-BY 4.0). Source code available from: https://github.com/lauracorbin/bbs_alspac_metabolon_datanote Archived code available from: 10.5281/zenodo.17198814 License: Creative Commons Attribution 4.0 International

## References

[1] Würtz P Kangas AJ Soininen P : Quantitative serum nuclear magnetic resonance metabolomics in large-scale epidemiology: a primer on -Omic Technologies. *Am J Epidemiol.* 2017;186(9):1084–1096. 10.1093/aje/kwx016 29106475 PMC5860146

[2] Pietzner M Stewart ID Raffler J : Plasma metabolites to profile pathways in noncommunicable disease multimorbidity. *Nat Med.* 2021;27(3):471–479. 10.1038/s41591-021-01266-0 33707775 PMC8127079

[3] Nicholson JK Holmes E Kinross JM : Metabolic phenotyping in clinical and surgical environments. *Nature.* 2012;491(7424):384–392. 10.1038/nature11708 23151581

[4] Fraser A Macdonald-Wallis C Tilling K : Cohort profile: the Avon Longitudinal Study of Parents and Children: ALSPAC mothers cohort. *Int J Epidemiol.* 2013;42(1):97–110. 10.1093/ije/dys066 22507742 PMC3600619

[5] Boyd A Golding J Macleod J : Cohort profile: the ‘children of the 90s’—the index offspring of the Avon Longitudinal Study of Parents and Children. *Int J Epidemiol.* 2013;42(1):111–127. 10.1093/ije/dys064 22507743 PMC3600618

[6] Northstone K Lewcock M Groom A : The Avon Longitudinal Study of Parents and Children (ALSPAC): an update on the enrolled sample of index children in 2019 [version 1; peer review: 2 approved]. *Wellcome Open Res.* 2019;4:51. 10.12688/wellcomeopenres.15132.1 31020050 PMC6464058

[7] Rogers CA Welbourn R Byrne J : The By-Band study: gastric bypass or adjustable gastric band surgery to treat morbid obesity: study protocol for a multi-centre randomised controlled trial with an internal pilot phase. *Trials.* 2014;15(1): 53. 10.1186/1745-6215-15-53 24517309 PMC3942168

[8] Rogers CA Reeves BC Byrne J : Adaptation of the By-Band randomized clinical trial to By-Band-Sleeve to include a new intervention and maintain relevance of the study to practice. *Br J Surg.* 2017;104(9):1207–1214. 10.1002/bjs.10562 28703939 PMC5519950

[9] By-Band-Sleeve Collaborative Group: Roux-en-Y gastric bypass, gastric banding, or sleeve gastrectomy for severe obesity: baseline data from the By-Band-Sleeve randomized controlled trial. *Obesity (Silver Spring).* 2023;31(5):1290–1299. 10.1002/oby.23746 37140395

[10] Cirulli ET Guo L Leon Swisher C : Profound perturbation of the metabolome in obesity is associated with health risk. *Cell Metab.* 2019;29(2):488–500. e2. 10.1016/j.cmet.2018.09.022 30318341 PMC6370944

[11] Harris PA Taylor R Thielke R : Research Electronic Data Capture (REDCap)--a metadata-driven methodology and workflow process for providing translational research informatics support. *J Biomed Inform.* 2009;42(2):377–381. 10.1016/j.jbi.2008.08.010 18929686 PMC2700030

[12] By-Band-Sleeve Collaborative Group: Roux-en-Y gastric bypass, adjustable gastric banding, or sleeve gastrectomy for severe obesity (By-Band-Sleeve): a multicentre, open label, three-group, randomised controlled trial. *Lancet Diabetes Endocrinol.* 2025;13(5):410–426. 10.1016/S2213-8587(25)00025-7 40179925 PMC7619233

[13] Hughes DA Taylor K McBride N : metaboprep: an R package for preanalysis data description and processing. *Bioinformatics.* 2022;38(7):1980–1987. 10.1093/bioinformatics/btac059 35134881 PMC8963298

[14] Fang S Wade KH Hughes DA : A multivariant recall-by-genotype study of the metabolomic signature of BMI. *Obesity (Silver Spring).* 2022;30(6):1298–1310. 10.1002/oby.23441 35598895 PMC9324973

