# Supplementary material for: Untargeted metabolomics data in the By-Band-Sleeve trial and ALSPAC: integrating clinical trial and population cohort data
Source: Wellcome Open Res. 2026 Mar 4;10:632. Originally published 2025 Nov 10. [Version 2] doi: 10.12688/wellcomeopenres.24917.2 (PMC12982984; doi:10.12688/wellcomeopenres.24917.2)
Supplement: Peer review history

# Reviewer report: Reviewer response for version 2

**DOI:** 10.21956/wellcomeopenres.28686.r149872

**Published:** 2026-03-17

**Authors:** Athanasios Pantelis

**Affiliations:** 1 Athens Medical Group, Athens, Greece

All reviewers' comments from version 1 have been adequately addressed.

 Reviewer 1 of version 2, Dr. Kehagias (U Patras) has raised two points that warrant consideration before final approval for indexing.

Are sufficient details of methods and materials provided to allow replication by others?

Yes

Is the rationale for creating the dataset(s) clearly described?

Yes

Are the datasets clearly presented in a useable and accessible format?

Yes

Are the protocols appropriate and is the work technically sound?

Yes

Reviewer Expertise:

metabolic bariatric surgery; surgical oncology; general surgery; trauma; systematic reviews; meta-analyses; artificial intelligence

I confirm that I have read this submission and believe that I have an appropriate level of expertise to confirm that it is of an acceptable scientific standard.

# Reviewer report: Reviewer response for version 2

**DOI:** 10.21956/wellcomeopenres.28686.r149976

**Published:** 2026-03-12

**Authors:** Dimitrios Kehagias

**Affiliations:** 1 University General Hospital of Patras, Rion, Patras, Greece

Thank you for the opportunity to review this manuscript. The authors present the results of metabolomics after analyzing two different datasets. Overall, the manuscript is well structured, with transparent methods and clear messages. I only have minor comments, that should be added mostly on the discussion:

 1. Regarding the metabolomic footprint of metabolic bariatric surgery (MBS), how do you believe this will contribute in clinical practice? Is the patient going to have a benefit if we measure these parameters and how exactly? Do they have a predictive role or does it guide towards personalized strategies?

 2. Recurrent weight regain represents a significant limitation of the long-term outcomes of metabolic bariatric surgery (MBS), particularly after sleeve gastrectomy, and remains an area of ongoing investigation. Given that the present study explores metabolomic alterations following MBS, it would be valuable to discuss whether metabolomic changes may occur at specific time points and potentially contribute to the prediction or understanding of recurrent weight gain.

 In this context, the authors may consider including and discussing the following study, which identified critical postoperative time points associated with long-term clinical response and recurrent weight gain after sleeve gastrectomy:

 [Ref 1]

 Integrating these findings could provide additional context regarding the temporal dynamics of postoperative changes and their potential association with long-term weight outcomes.

Are sufficient details of methods and materials provided to allow replication by others?

Yes

Is the rationale for creating the dataset(s) clearly described?

Yes

Are the datasets clearly presented in a useable and accessible format?

Yes

Are the protocols appropriate and is the work technically sound?

Yes

Reviewer Expertise:

Metabolic Bariatric Surgery

I confirm that I have read this submission and believe that I have an appropriate level of expertise to confirm that it is of an acceptable scientific standard.

## References

[1] : Critical Time Points for Assessing Long-Term Clinical Response After Sleeve Gastrectomy—A Retrospective Study of Patients with 13-Year Follow-Up. *Obesity Surgery* .2025;35(2) : 10.1007/s11695-024-07659-7 571-581 10.1007/s11695-024-07659-7 39760990

# Reviewer report: Reviewer response for version 1

**DOI:** 10.21956/wellcomeopenres.27447.r144196

**Published:** 2026-01-19

**Authors:** Athanasios Pantelis

**Affiliations:** 1 Athens Medical Group, Athens, Greece

This Data Note describes untargeted serum metabolomics generated using the Metabolon Discovery HD4 platform in two populations: the ALSPAC “Focus at 30” cohort and participants from the By-Band-Sleeve (BBS) bariatric trial/sub-study. The authors provide transparent documentation of sample provenance, batch randomization, preprocessing and QC using *metaboprep*, including the exclusion of a clearly aberrant site-specific cluster, and validate the dataset through comparison with clinical chemistry and replicate analyses.

 The dataset is valuable and largely well documented. The main limitations are not technical, but relate to **usability for secondary analysts**, particularly clearer guidance on cohort harmonization, batch/site effects, and metadata completeness.

 As per my standard practice, I attempted to use the respective guidelines that are approrpiate for this kind of research. Since there is paucity of relevant EQUATOR guidelines, I used the FAIR Data Principles (Findable, Accessible, Interoperable, Reusable) for this Data Note.

**MAJOR POINTS**

1. **Site-based exclusion needs clearer documentation**
  The exclusion of 142 samples from one BBS site is reasonable and supported by PCA, but the manuscript should explicitly state:
  - the criteria used to define the outlier cluster;
  - whether root causes (handling, shipment, batch position) were investigated;
  - how downstream users should treat site effects in pooled analyses.
2. **User guidance for pooled analyses is insufficient**
  Given the heterogeneity between ALSPAC and BBS, the manuscript should include a short “Recommended use” section covering:
  - whether batch/plate/site covariates should be modeled;
  - cautions when combining cohorts;
  - suggested normalization or adjustment strategies.
3. **Metadata for reuse should be strengthened**
  To fully meet FAIR principles, users need clearer access to:
  - a data dictionary (variables, units);
  - missingness codes;
  - whether released values are raw or transformed;
  - dataset and pipeline versioning.

**MINOR POINTS**

- The abstract should explicitly report final sample size and metabolite count after QC.
- Clarify whether metabolomics and clinical chemistry measurements were taken from the same blood draw and fasting state.
- Avoid language implying clinical equivalence between untargeted MS and routine biochemistry.
- Ensure bariatric terminology (where relevant) aligns with current standards (e.g. *metabolic bariatric surgery* rather than legacy terms).

Overall Recommendation

**MINOR REVISION**

 This is a solid, transparent Data Note describing a high-quality metabolomics resource. With clearer documentation of QC decisions and stronger guidance for secondary users, it will be a useful and reusable dataset for the field.

Are sufficient details of methods and materials provided to allow replication by others?

Yes

Is the rationale for creating the dataset(s) clearly described?

Yes

Are the datasets clearly presented in a useable and accessible format?

Yes

Are the protocols appropriate and is the work technically sound?

Yes

Reviewer Expertise:

metabolic bariatric surgery; surgical oncology; general surgery; trauma; systematic reviews; meta-analyses; artificial intelligence

I confirm that I have read this submission and believe that I have an appropriate level of expertise to confirm that it is of an acceptable scientific standard, however I have significant reservations, as outlined above.

# Response

**Published:** 2026-02-09

**Authors:** Laura Corbin

**Affiliations:** University of Bristol, UK

*The authors would like to thank the reviewer for their constructive comments.*   MAJOR POINTS

1. Site-based exclusion needs clearer documentation

The exclusion of 142 samples from one BBS site is reasonable and supported by PCA, but the manuscript should explicitly state:

1. the criteria used to define the outlier cluster;

 
*Response: The cut offs used to identify the samples to exclude has been added to the manuscript. See also Script 10 in the GitHub repository.*  

1. whether root causes (handling, shipment, batch position) were investigated;

 
*Response: Further details of the investigation of possible causes for the outlying cluster have been added. See also Script 10 in the GitHub repository. After this work, the same samples were sent for NMR analysis (data note in process). We included duplicates for the outliers to help determine if the issue was with the specific sample aliquot or the whole source sample. It seems most likely that the entire sample was affected leading us to believe the issue was with sample handling prior to samples being received by the lab at University of Bristol. *  

1. how downstream users should treat site effects in pooled analyses. User guidance for pooled analyses is insufficient

 
*Response: Text has been added suggesting that several technical covars, including site, be accounted for in analyses of BBS data (see new ‘Recommended use’ section). *  

1. Given the heterogeneity between ALSPAC and BBS, the manuscript should include a short “Recommended use” section covering:
  1. whether batch/plate/site covariates should be modeled;
  2. cautions when combining cohorts;
  3. suggested normalization or adjustment strategies.

 
*Response: See newly added ‘Recommended use’ section which brings together new text and text moved from elsewhere in the manuscript.* *We have designed the experiment such that technical batch effects relating to MS data generation have been eliminated by analysing samples from the two studies in the same Metabolon project and by balancing samples across experimental batches. Therefore, any remaining structure will be due to cohort-specific effects either to do with differences in processes (e.g. sample collection protocols) or participants (e.g. age). The nature of the study design is such that we do not think it is possible to harmonize/normalise data without removing some of the characteristics of interest. For example, if you normalise within studies, then our ability to investigate the BMI effect on metabolites, for example, would be greatly compromised. Therefore, our expectation would be that researchers use the combined dataset alongside other complementary study designs to answer questions of interest.*  

1. Metadata for reuse should be strengthened. To fully meet FAIR principles, users need clearer access to:
  1. a data dictionary (variables, units);

 
*Response: We have added three data dictionaries (one per dataset) to the extended data in the OSF project).*  

1.
  1. missingness codes;

 
*Response: We have added further description of the datasets in the ‘Data and code availability section.’*  

1.
  1. whether released values are raw or transformed;

 
*Response: We have added further description of the datasets in the ‘Data and code availability section.’*  

1.
  1. dataset and pipeline versioning.

 
*Response: We have added version numbers to the datasets and code (which has now iterated from v1.0-alpha as it was at first submission to v2.0-alpha).*   MINOR POINTS

1. The abstract should explicitly report final sample size and metabolite count after QC.

 
*Response: This information is in the Results section of the Abstract and has been updated following a minor change to the data preparation pipeline (specifically the removal of metabolites observed in <5 individuals to protect participant anonymity). *  

1. Clarify whether metabolomics and clinical chemistry measurements were taken from the same blood draw and fasting state.

 
*Response: This information has been added to the ‘Sample collection’ section of the Methods.*  

1. Avoid language implying clinical equivalence between untargeted MS and routine biochemistry.

 
*Response: We have clarified the value of the comparison to this work by adding a sentence to the ‘Discussion.’*  

1. Ensure bariatric terminology (where relevant) aligns with current standards (e.g. metabolic bariatric surgery rather than legacy terms).

 
*Response: We have updated the terminology as suggested.*

# Reviewer report: Reviewer response for version 1

**DOI:** 10.21956/wellcomeopenres.27447.r140934

**Published:** 2026-01-05

**Authors:** ABDULZAHRA HUSSAIN, ALAA AL WADEES

**Affiliations:** 1 Jabir ibn Hayyan Medical University Faculty of Medicine (Ringgold ID: 480179), Kufa, Najaf Governorate, Iraq; 2 Minimal access unit.General surgery, Homerton University Hospital (Ringgold ID: 156566), London, England, UK

**Untargeted metabolomics data in the By-Band-Sleeve trial and ALSPAC: integrating clinical trial and population cohort data**

 Dear Editor in Chief :

 Thank you for involving me. I have read with interest the article, and I have put down my comments.

1. **The study has Clear Scope and Purpose: **The introduction concisely states an overview of MS (mass‑spectrometry) metabolite data from two cohorts (the ALSPAC Focus‑at‑30 clinic and the By‑Band‑Sleeve trial [BBS] participants).Pre-analytical processing, QC, participant characteristics, data validation — giving confidence in thoroughness.
2. **Authors showed Transparency about Data Quality & Limitations**:Overall concordance between MS and standard clinical‑chemistry assays is “good” but there are “some outlying samples.”Specific issues are highlighted (e.g., lowest cross-platform correlation for total cholesterol) and plausible reasons (different lipid entities, different sensitivity/specificity).The readers are cautioned to consider potential outliers— a very appropriate caveat.
3. **The study demonstrates the Value of the Dataset**: The dataset provides: combining metabolomics with rich phenotypic data from ALSPAC; exploring effects of bariatric surgery from BBS; comparing “healthy population” vs intervention cohorts; studying metabolic changes due to weight loss.The study provides numbers (1951 samples total, breakouts for joint, BBS‑only, ALSPAC‑only) that improve reproducibility and transparency.
4. **Open‑Science / Data Access Guidance**: Including instructions on how to request access to ALSPAC data and conditions for accessing BBS data is helpful and supports reusability. The mention of a project code (B4132) and a link to publicly available supplementary information (on OSF) support openness
5. **Abstract:** A non-structured abstract was provided ,Q: please consider structured abstract:Background, methods, results, conclusions?
6. **Introduction:**Q: The following statement can be removed and moved to the bottom of the introduction section.[Here we present a description of metabolomics data generated using an untargeted mass-spectrometry approach in two studies: (1) the Avon Longitudinal Study of Parents and Children (ALSPAC) - a healthy, general population; and (2) the By-Band-Sleeve trial (BBS) - a randomised controlled trial (RCT) of bariatric surgery plus a non-randomised observational sub-study.]
7. **Methods: **A. Are the data related to all adults of **Avon Longitudinal Study of Parents and Children (ALSPAC), or some of it taken from 7 years old ? **Q: Please clarify Q: If you have included data of 7 years old , then what is the rational of comparing these data to the BBS adult data?
8. **Metabolites data acquisition:**(The mass spectrometry platform used by Metabolon to measure metabolites provides semi-quantitative abundance data rather than absolute quantifications; therefore, the data are relative and cannot be combined across studies at the individual level after metabolomics analysis. Having ALSPAC and BBS samples analysed within the same experiment allows the data to be analysed together)If we cannot quantify the data and draw a solid conclusions, then what is the value and the impact of the study on the current practice?How is the study changing practice ?

----------------------------------------------------------------------------------------------------------------

**9. Discussion:**

**Issues, Ambiguities, and Areas for Improvement**

 While the draft is good in many respects, there are several areas — some small, some more substantial — that could benefit from revision, clarification, or expansion .

**1. Structure and Flow**The text, as given, feels somewhat **dense** and **stream-of-consciousness** in places. It may be difficult for readers to follow the logic across paragraphs. Consider breaking it into clearly labelled subsections.Relatedly, some paragraphs try to do too many things (e.g., discuss validation results, then jump to utility, then to limitations).

**2. Lack of Detail in Some Methods / QC Steps:**You mention “pre-analytical data processing, QC, participant characteristics and data validation,” but you do **not** summarise **how** QC was done (what thresholds, what criteria for outlier detection, whether batch effects/normalisation/imputation were used, etc.). For a data note, readers will expect at least a summary of key QC steps. Without that, it is hard to assess data robustness.Similarly, “data validation” is mentioned, but only one example (correlation of metabolites common to MS and clinical assays) is given. Regarding the comment "some metabolites were not consistently measured across all concentrations,” it’s vague. You should specify how many metabolites, what proportion, whether missingness was random or concentration‑dependent, and how users should interpret/handle that.

**3. Ambiguous Statements: **Example: “We found good overall concordance, although there were some outlying samples.” What does “good” mean? Perhaps give median correlation or range? The reader will want quantitative metrics.“readers may want to check that any metabolites of interest … have good consistency.” This is sensible but rather weak as guidance. Instead, you could provide a table (or summary) of which metabolites passed QC / consistency thresholds, and which are more variable.The phrase “very different studies comprising different populations and delivered according to different protocols, therefore the combined data should be used with caution.” This is true — but perhaps underplays the risk. Combining data from heterogeneous studies may introduce substantial batch or cohort effects. Without formal harmonisation or adjustment, combining may lead to misleading inferences.

**4. Limitations Underrepresented / Bias Concerns**

 Some limitations, but some additional potential biases and pitfalls deserve mention:

- **Selection bias in ALSPAC subsample**: participants in this dataset are those who “attended the age 30 clinic first” — this may introduce bias (e.g., healthier, more motivated, more compliant individuals). You mention it, but the implications should be more explicitly discussed (e.g., how this may limit generalizability even within ALSPAC).
- **Missing longitudinal data in BBS cohort**: the fact that many baseline samples lack post-surgery follow‑up is stated, but the impact on power / ability to detect within‑individual changes should be more deeply discussed.
- **Ancestry limitation**: participants are predominantly White / European ancestry. It would be helpful to quantify roughly what percent (if known), or at least note the absence of diversity and implications for generalizability.
- **Potential batch/cohort effects**: combining data from two separate studies with different collection protocols, processing pipelines, and participant characteristics almost always introduces batch effects. The draft says “should be used with caution,” but does not describe any harmonisation or adjustment. Without harmonisation methods or clear QC criteria for cross-study analyses, downstream analyses (especially across cohorts) may be confounded.

**5. Tone & Recommendations— Too Soft / Vague**

- The recommendations to “consider outliers … in a way suitable to your research question” or “apply additional QC procedures and validation analyses” are correct, but a bit too soft. For a indexed data note, reviewers/readers will expect stronger guidance or at least a suggested recommended practice. For example:
  - Propose concrete QC steps or thresholds (e.g., exclude samples with more than X% missing, or metabolites with CV > Y, or correlate poorly with clinical assays).
  - Recommend normalisation (log‑transform, quantile, etc.), batch‑effect correction (if combining across cohorts), and handling of missing data/outliers.
  - Suggest documenting and reporting any deviations/exclusions in subsequent analyses.

**6. Minor Editorial / Clarity / Grammar / Formatting Issues:**There are some typographical and formatting issues (e.g., “god good storage capacity,” Some sentences are long and complex; breaking them up would help readability.When referring to cohorts or studies (e.g., ALSPAC, BBS), consider defining them at first mention (full name, abbreviation).It might help to include a small table (or refer to one) summarizing sample counts, basic demographics (age / sex / ancestry), QC pass/fail summary, etc.

Are sufficient details of methods and materials provided to allow replication by others?

Yes

Is the rationale for creating the dataset(s) clearly described?

Yes

Are the datasets clearly presented in a useable and accessible format?

Yes

Are the protocols appropriate and is the work technically sound?

Yes

Reviewer Expertise:

Clinical research in surgery , T2DM,obesity

We confirm that we have read this submission and believe that we have an appropriate level of expertise to confirm that it is of an acceptable scientific standard.

# Response

**Published:** 2026-02-09

**Authors:** Laura Corbin

**Affiliations:** University of Bristol, UK

*The authors would like to thank the reviewer for their constructive comments.*

1. Abstract: A non-structured abstract was provided ,Q: please consider structured abstract: Background, methods, results, conclusions?

 
*Response: Abstract has been structured and edited accordingly.*  

1. Introduction:Q: The following statement can be removed and moved to the bottom of the introduction section.[Here we present a description of metabolomics data generated using an untargeted mass-spectrometry approach in two studies: (1) the Avon Longitudinal Study of Parents and Children (ALSPAC) - a healthy, general population; and (2) the By-Band-Sleeve trial (BBS) - a randomised controlled trial (RCT) of bariatric surgery plus a non-randomised observational sub-study.]

 
*Response: We moved this sentence to the Abstract as we thought it worked better there given the new structure of that section.*  

1. Methods: A. Are the data related to all adults of Avon Longitudinal Study of Parents and Children (ALSPAC), or some of it taken from 7 years old ? Q: Please clarify Q: If you have included data of 7 years old , then what is the rational of comparing these data to the BBS adult data?

 
*Response: All the data were generated from samples collected from ALSPAC participants during the age 30-year clinic.*  

1. Metabolites data acquisition:(The mass spectrometry platform used by Metabolon to measure metabolites provides semi-quantitative abundance data rather than absolute quantifications; therefore, the data are relative and cannot be combined across studies at the individual level after metabolomics analysis. Having ALSPAC and BBS samples analysed within the same experiment allows the data to be analysed together)If we cannot quantify the data and draw a solid conclusions, then what is the value and the impact of the study on the current practice?How is the study changing practice ?

 
*Response: We are unsure exactly what the reviewer means here but have edited this section of the manuscript to improve clarity and hope this has addressed this query.*  

1. While the draft is good in many respects, there are several areas — some small, some more substantial — that could benefit from revision, clarification, or expansion.

 

1.
  1. Structure and Flow: The text, as given, feels somewhat dense and stream-of-consciousness in places. It may be difficult for readers to follow the logic across paragraphs. Consider breaking it into clearly labelled subsections.Relatedly, some paragraphs try to do too many things (e.g., discuss validation results, then jump to utility, then to limitations).

 
*Response: We reordered results to put participant characteristics first which corresponds better with the order in the Methods. To improve readability, we made sure all subheadings in the Methods had corresponding sections in the Results. Finally, a ‘Recommended use’ section has been added to the Discussion to bring together guidance (content that was previously scattered through the paper).*  

1.
  1. Lack of Detail in Some Methods / QC Steps: You mention “pre-analytical data processing, QC, participant characteristics and data validation,” but you do not summarise how QC was done (what thresholds, what criteria for outlier detection, whether batch effects/normalisation/imputation were used, etc.). For a data note, readers will expect at least a summary of key QC steps. Without that, it is hard to assess data robustness.

 
*Response: All this information is contained within the accompanying GitHub code and OSF project. We have added a statement at the end of the QC methods signposting readers to these resources.*  

1.
  1. Similarly, “data validation” is mentioned, but only one example (correlation of metabolites common to MS and clinical assays) is given. Regarding the comment "some metabolites were not consistently measured across all concentrations,” it’s vague. You should specify how many metabolites, what proportion, whether missingness was random or concentration‑dependent, and how users should interpret/handle that.

 
*Response: There are three investigations under the ‘Data validation’ subheading – (1) Comparison with clinical chemistry; (2) Comparison to previously generated BBS data; (3) Test of batch normalization procedure. Regarding the observations comparing 2021 and 2023 data generation, whilst some metabolites did not show the same consistency across the full concentration, with >1200 metabolites in the dataset and this assessment being essentially qualitative (based on Bland-Altman plots) it is not practical to provide advice on a per-metabolite basis. Furthermore, we cannot say which of the measures are more accurate so would not advocate removing specific data points. Our aim is to raise awareness of the issue and encourage data users to check metabolites of interest as they draw conclusions from their own results.*  

1.
  1. Ambiguous Statements: Example: “We found good overall concordance, although there were some outlying samples.” What does “good” mean? Perhaps give median correlation or range? The reader will want quantitative metrics.“readers may want to check that any metabolites of interest … have good consistency.” This is sensible but rather weak as guidance. Instead, you could provide a table (or summary) of which metabolites passed QC / consistency thresholds, and which are more variable.

 
*Response: The data to support this statement in the discussion can be found in the Results section ‘Comparison with clinical chemistry’ and in Supplementary Figure 1 and Figure 3. As above, with this type of data, providing metabolite-level criteria for inclusion/exclusion beyond that already done (using metaboprep) is challenging. Often this level of QC is context specific and therefore we have applied a relatively inclusive QC procedure and invite data users to apply further QC and/or investigations as part of their work.*  

1.
  1. The phrase “very different studies comprising different populations and delivered according to different protocols, therefore the combined data should be used with caution.” This is true — but perhaps underplays the risk. Combining data from heterogeneous studies may introduce substantial batch or cohort effects. Without formal harmonisation or adjustment, combining may lead to misleading inferences.

 
*Response: Here we have designed the experiment such that technical batch effects relating to MS data generation have been eliminated by analysing samples from the two studies in the same Metabolon project and by balancing samples across experimental batches. Therefore, any remaining structure will be due to cohort-specific effects either to do with differences in processes (e.g. sample collection protocols) or participant characteristics (e.g. age). Therefore, while we agree caution is needed, provided analyses are carried out and interpreted with this knowledge, ‘misleading inferences’ should be avoidable.*  

1. Limitations Underrepresented / Bias Concerns. Some limitations, but some additional potential biases and pitfalls deserve mention:
  1. Selection bias in ALSPAC subsample: participants in this dataset are those who “attended the age 30 clinic first” — this may introduce bias (e.g., healthier, more motivated, more compliant individuals). You mention it, but the implications should be more explicitly discussed (e.g., how this may limit generalizability even within ALSPAC).

 
*Response: We have added a sentence explaining the possible consequences of this selection strategy and added a recommendation for data users to check for generalisability as part of their analyses.*  

1.
  1. Missing longitudinal data in BBS cohort: the fact that many baseline samples lack post-surgery follow‑up is stated, but the impact on power / ability to detect within‑individual changes should be more deeply discussed.

 
*Response: We have added more detail about this to the ‘Strengths and limitations’ and ‘Recommended use’ sections.*  

1.
  1. Ancestry limitation: participants are predominantly White / European ancestry. It would be helpful to quantify roughly what percent (if known), or at least note the absence of diversity and implications for generalizability.

 
*Response: This information is provided in Table 2 for ALSPAC and Table 3 for BBS. There is a statement in the ‘Strengths and limitations’ section acknowledging the lack of generalisability beyond the White European population.*  

1.
  1. Potential batch/cohort effects: combining data from two separate studies with different collection protocols, processing pipelines, and participant characteristics almost always introduces batch effects. The draft says “should be used with caution,” but does not describe any harmonisation or adjustment. Without harmonisation methods or clear QC criteria for cross-study analyses, downstream analyses (especially across cohorts) may be confounded.

 
*Response: The nature of the study design is such that we do not think it is possible to harmonize/normalise data across cohorts without removing some of the characteristics of interest. For example, if you normalise within studies, then our ability to investigate, for example, the BMI effect on metabolites would be greatly compromised. As mentioned above, the data collection was carried out in such a way as to minimise the technical batch effects. Our recommendation would be to use the combined dataset alongside other complementary study designs to answer questions of interest.*  

1. Tone & Recommendations— Too Soft / Vague
  1. The recommendations to “consider outliers … in a way suitable to your research question” or “apply additional QC procedures and validation analyses” are correct, but a bit too soft. For a indexed data note, reviewers/readers will expect stronger guidance or at least a suggested recommended practice. For example:
    1. Propose concrete QC steps or thresholds (e.g., exclude samples with more than X% missing, or metabolites with CV > Y, or correlate poorly with clinical assays).
    2. Recommend normalisation (log‑transform, quantile, etc.), batch‑effect correction (if combining across cohorts), and handling of missing data/outliers.
    3. Suggest documenting and reporting any deviations/exclusions in subsequent analyses.

 
*Response: In our opinion, many of the pre-analytical processing decisions that you mention need to be made in the context of the specific analysis being done. This is why we have not applied any further transformations, imputations, etc, to the data. We have added some additional guidance to the ‘Recommended use’ section and referenced a previous manuscript where we have discussed these issues at length (Hughes et al 2022). *  

1. Minor Editorial / Clarity / Grammar / Formatting Issues:
  1. There are some typographical and formatting issues (e.g., “god good storage capacity,”

 
*Response: Apologies, we could not find this error in the text but have proofread the revised version. *  

1.
  1. Some sentences are long and complex; breaking them up would help readability.

 
*Response: We have broken up long sentences where possible.*  

1.
  1. When referring to cohorts or studies (e.g., ALSPAC, BBS), consider defining them at first mention (full name, abbreviation).

 
*Response: We have defined the abbreviations in the intro of the Abstract, in the Introduction and in the subheadings within the Methods. *  

1.
  1. It might help to include a small table (or refer to one) summarizing sample counts, basic demographics (age / sex / ancestry), QC pass/fail summary, etc.

 
*Response: Participant characteristics are provided* *in Table 2 for ALSPAC and Table 3 for BBS. A summary of sample/feature exclusions is provided in Figure 1.*
